# Computational dynamic approaches for temporal omics data with applications to systems medicine

**DOI:** 10.1186/s13040-017-0140-x

**Published:** 2017-06-17

**Authors:** Yulan Liang, Arpad Kelemen

**Affiliations:** 1 Department of Family and Community Health, University of Maryland, Baltimore, MD 21201 USA; 2 Department of Organizational Systems and Adult Health, University of Maryland, Baltimore, MD 21201 USA

**Keywords:** Temporal omics data, Dynamic approaches, Trajectory prediction, Causal network, Systems medicine, Computational dynamic approaches for temporal omics data with applications to systems medicine

## Abstract

Modeling and predicting biological dynamic systems and simultaneously estimating the kinetic structural and functional parameters are extremely important in systems and computational biology. This is key for understanding the complexity of the human health, drug response, disease susceptibility and pathogenesis for systems medicine. Temporal omics data used to measure the dynamic biological systems are essentials to discover complex biological interactions and clinical mechanism and causations. However, the delineation of the possible associations and causalities of genes, proteins, metabolites, cells and other biological entities from high throughput time course omics data is challenging for which conventional experimental techniques are not suited in the big omics era. In this paper, we present various recently developed dynamic trajectory and causal network approaches for temporal omics data, which are extremely useful for those researchers who want to start working in this challenging research area. Moreover, applications to various biological systems, health conditions and disease status, and examples that summarize the state-of-the art performances depending on different specific mining tasks are presented. We critically discuss the merits, drawbacks and limitations of the approaches, and the associated main challenges for the years ahead. The most recent computing tools and software to analyze specific problem type, associated platform resources, and other potentials for the dynamic trajectory and interaction methods are also presented and discussed in detail.

## Introduction

Recent advancement in the omics fields (i.e., genomics, transcriptomics, variomics, proteomics, metabolomics, and interactomics) and the associated technologies (from microarray. RNA sequencing, whole genome sequences (WGS), mass spectrometry (MS)) have provided huge amount of information for delineating the roles of biological entities (i.e., gene mutants, DNA methylations, metabolites) in complex diseases and biological system states for the human organisms [1–7]. On the other hand, the systems and precision medicine known as P4 medicine - Predictive, Preventive, Personalized and Participatory, have been hot topics given the amount of big omics data and knowledge accumulated in the past decades from translational medicine and human genomic/proteomic research [8–11]. In systems medicine, the human organism is envisioned as a system of systems or network of networks, which is hierarchically and biologically organized from genomic/proteomic to molecular, to cellular, to organ, to individual human body, to social/environmental human systems. At each level/scale, those are dynamically embedding each other (as opposed to being static) [8–11].

Despite considerable computational and statistical efforts over the decades with thousands of computational tools, algorithms and models developed ranging from single model to multi-level (such as meta-frame), the key computational challenges of system medicine remains: how to best mine and learn the continuing arrival of big omics data given thousands of interacting entities (e.g., genes or proteins) with relatively weak or small accumulative effects over time on health conditions or diseases [12–14]. The overwhelming number of confounded traits or highly correlated phenotypes with the unavoidable measurement noises makes the integrations even harder, not just metadata or models, but also the results. Moreover, the different topological characteristics of the biological omics data require different sets of algorithms and models (i.e., supervised versus unsupervised; generative or discriminative) for deriving meaningful interpretable relationships. Nevertheless, omics data aggregations, linkage, curation, validation issues from diverse platforms, software outputs, inconsistent data standardizations make clinical implementations harder [15–17]. In addition, analyzing and processing too much combined large data may cause over fitting issues, too complex unstable model, sacrificing predictive accuracy.

**Fig. 1:**
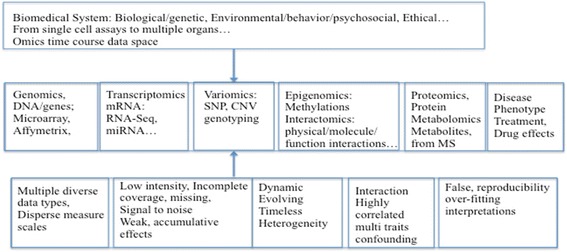
Various Omics data types and challenges

From the clinical or biomedical perspective, the challenge issue is the reliability for avoiding false discovery, and reproducibility across different patient cohorts and the associated biological interpretability of the findings. These are all crucial in order to extract fully confirmed actionable knowledge for systems medicine and P4 solutions. But the evolving, heterogeneous and dynamic information with low intensity signals with respect to noise from omics technologies make the key drivers led to complex diseases difficult to characterize. Fig. 1 display the various omics data types and associated challenges. Time-course or temporal omics (i.e., genomic/proteomic/metabolites) experiments are often used to measure and study dynamic biological and medical systems. Knowing when or whether a biological entities including genes or proteins are expressed or regulated, and how one interacts with others can provide a strong clue of their biological roles and potential causality for disease conditions that may have therapeutic implication, i.e., not treated versus combination of treatments; recurring disease patterns, disease subtypes, and key regulatory pathways of drug effects [18–22].

To tackle those dynamic, interacting, hidden but valuable biomedical information, various analytical tools ranging from single level to more sophisticated hybrid data mining, machine learning tools, and advanced statistical models are needed, especially the advanced approaches for causal network inferences and dynamic trajectory predictions for drug and disease responses [5, 6, 8–11]. This paper focuses on the various trajectory and interaction approaches for temporal omics data, ranging from single level to multilevel network/cloud computing. These approaches can be either model based (statistical, mathematical, neural network (NN)) or algorithm based (machine learning or data mining) or hybrid ensemble approaches (i.e. with knowledge integration). The examples and recently developed computing tools/resources for comparing various trajectory and interaction methods regarding the merits and drawbacks use the same data sets or different data sets are presented. More applications to pathway, regulations, function, and integrative meta-analysis for various human health, conditions, and diseases are given special attention. Other potentials for future directions (intelligent approaches with deep learning, automatic reasoning; consensus predictions with boosts and bagging) are discussed.

### Computational apparoches for temporal experiments

**Fig. 2:**
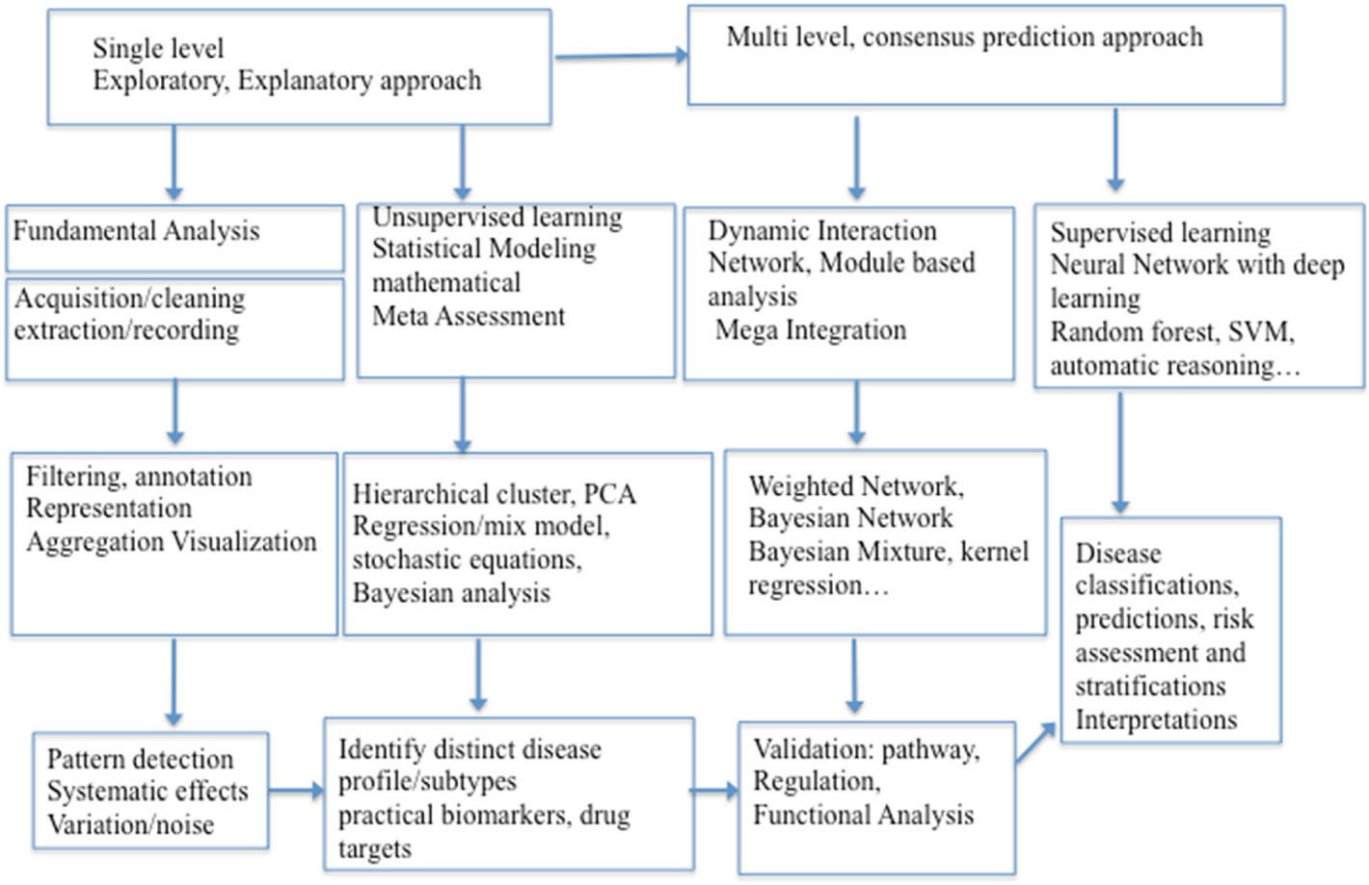
Computational approaches for omics data from single level to multi-level, network/pathway and clinical outcomes

To process and model the temporal omics data, several layer/levels analyses could be applied to meet the needs of the state-of-the-art omics data in order to overcome the challenges. Fig. 2 provides an overview of various computational methods starting from low-level fundamental analysis to immediate, then to advanced analysis. Fundamental analyses include data acquisition, noise filtering, system effect detection, etc. to ensure the quality of the data and outcomes. Immediate analyses include different data reduction techniques for high dimensionality issue, i.e., statistical variable selection/screening, machine-learning algorithm with feature extractions, and mathematical modeling (i.e., optimization). For instance, using supervised learning with wrapper methods for feature/gene selections, the significantly differentially expressed gene can be identified out of thousands of genes.

**Fig. 3:**
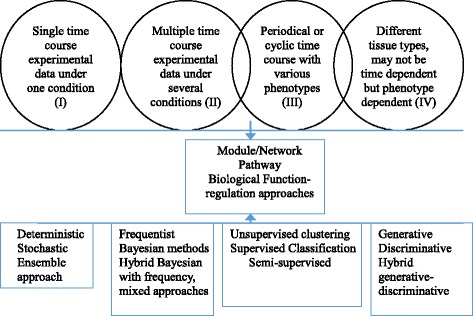
Venn Diagram of general computational framework for high dimensional time course Omics data for System and precision medicine

One of the goals of modeling temporal omics data is to infer and predict the biological networks and interactions and for further causal, pathway, function and integrative analysis. The advanced level analysis is the focus of the paper, which includes dynamic trajectory, interactions, network/module based modeling, and knowledge/data integrations with pathway, regulatory and function analysis. Figure 3 provides a Venn diagram of general dynamic computational framework for different types of high dimensional time course omics data. All layers/levels of analyses are critical steps when modeling the high dimensional omics data, especially when time dimensions are added with various types of time course experiment data. As the omics data continues to grow, the analytical scheme needs to be switched from correlation or module, pattern based approaches towards to network, module based, then causal, pathway, function integrative based (see Fig. 3: outside circle towards to the center) for actionable P4 solution. Table 1 summarizes an overview of the comparison of the various dynamic modeling approaches for temporal omics data from computational perspective, which are presented in details next.

**Table 1:** Comparison of the dynamic modeling approaches for temporal omics data, the detailed methods, mining tasks, and type of problems, examples, and related references

General approaches	Examples	Type of problems, tasks	Important features and functions	Some Reference
Math based Deterministic, static Stochastic, dynamic	Differential equations, Fourier transform, topology based matrix factorization Stochastic differential equations, Gaussian graphical models, Probabilistic Boolean networks State space model and or hidden Markov model, Markov random fields	Parameter/rate estimations, network inference, prediction, time course (I-III) Dynamic parameter estimations transition process Causal or non-causal temporal relationships	Fixed, stable parameter, structure estimation, time invaried, non-causal Direct relationship, Nonlinear or linear. Probabilistic time varied, Nonlinear or linear Direct or indirect relationship time course (I-III)	[23–30] [36–40] [31–35] [41, 42] [43]
Statistical based Frequentist/classical Bayesian methods	Regression vector autoregressive (VAR) models, Curve fitting, spline methods, Granger causality Bayesian models (linear or nonlinear model), growth model	Parameter estimations, predictions, hypothesis testing, biomarker/target identifications Heterogeneity discovery	Explanatory relationship without prior knowledge, pure data based time course (I-III) or phenotype dependent (IV) With prior or empirical Knowledge, probabilistic	[35, 44, 45] [46] [41, 42, 47–57]
Computer sciences based Machine learning, data mining discriminative generative Neural network	Unsupervised: Distance or correlation based Supervised classification with wrapper Feedback Forward NN, time recurrent NN, convolution NN, Bayesian NN	Subtypes, modular, and heterogeneity discovery, Pattern discovery and identification Dynamic changes and trajectories Complex relationship, structure	Time course (I-III) or phenotype dependent (IV) Without knowing the outcome, classes, Defined outcomes/classes conditional joint analysis Time varied or invariaed Nonlinear or linear Direct or indirect relationship, Explanatory or predictive time course (I-III) or phenotype dependent (IV)	[26–29] [58–66] [68–75]
Interactions and network, pathway function based	Predictions, integrated with public databases	phenotype dependent (IV), Graphic based Causal hypothesis	Direct or indirect relationship, Nonlinear or linear integrated with public databases interactive through manually or automate	[89–109, 116, 162] [83–85] [86] [87]

### Mathematical modeling: discrete static versus continuous dynamic approaches

The synergistic system formalism is a static differential equation based deterministic approach that has been applied to genetic, immune and biochemical network data [23–25]. Nonlinear discrete dynamical systems also have been developed and applied for temporal data analysis [25–28]‬. As an example, discrete Boolean networks are developed as probabilistic models of gene regulatory interactions. The corresponding networks are able to cope with uncertainty in order to discover the relative sensitivity of gene-gene interactions [26, 27]. These systems are non-linear and many advanced computational algorithms such as genetic algorithms and linear programming have been implemented for time courses of gene expression. Such types of deterministic interaction models can potentially provide valuable quantitative and mechanistic descriptions of gene activities that may be mediated by drugs and pharmacological agents. However, these traditional mathematical models have not incorporated the stochastic nature of biological process; the time delay or order information and they often treat the biological parameters as fixed values and model them in deterministic ways involved in the estimations.

Singular value decomposition has also been developed for modeling the dynamics of microarray experimental data through matrix decomposition and eigenvalue analysis [29, 30]. The difficulties of these methods are the estimation of the dimensionality of large matrix with ill-posed problems due to large p small n. Dynamic matrix-variate graphical models have demonstrated promising results for dynamic genetic network constructions, have applied for identification of age-related patterns in a public, prefrontal cortex gene expression dataset [31–35]. Topology network and graph based multi-scale approaches decompose the network into subsystems (such as modules and pathways) utilizing various metric measures [7], which could be further used for predicting the specific functions or phenotypes.

Stochastic paradigm treats the dynamic process of temporal change as a stochastic process and describes it as a probability system in time with uncertainty [36–40]. Examples of stochastic processes are Gaussian process, Markov process, and point process. The advantage of using a stochastic process is that it accounts for the temporal information in the model. The drawback is that it makes some assumptions to model the process, which may not be valid. Chen and colleagues (2005) combine the stochastic process with differential equation and developed a stochastic differential equation model for quantifying transcriptional regulatory network in Saccharomyces cerevisiae [39].

State space model (dynamic linear models) and hidden Markov model are two important applications of statistical models combined with stochastic process techniques. State space model combines the stochastic process with the observation data model uniformly to model a continuous process for capturing the change of gene states [41]. Hidden Markov model can be used to model the gene activity systems in which the gene states are unobservable, discrete, but can be represented by a state transition structure determined by the state parameters and the state transition matrix while processing the patterns over time [42].

State space models have greater flexibility in modeling non-stationary and nonlinear short time course data and were implemented and applied to genomic studies [41]. However, some existing algorithms for these models were based on standard Kalman filter methods, which rely on the linear state transitions and Gaussian errors. Perrin et al. used a penalized likelihood maximization implemented through an extended version of EM algorithm to learn the parameters of the model [43]. Rangel, et al. used classical cross-validations and Bootstrap techniques and Beal et al. used variation approximations with linear time invariant Gaussian setting for constructions of the regulatory network [41].

### Statistical approaches: frequentist versus Bayesian methods

The choice of statistical modeling approaches for temporal omics data depends on the features and types of the data (univariate (I), multivariate (II), cycling (III), phenotype dependent (IV), Fig. 3). The statistical approaches also depend on the scale of the observed outcomes (continuous, discrete: ordinal, binary) and the structure of the balanced or unbalanced data (i.e., diseases type with much more sample than the comparison sample). The associated analysis can be 1) analysis of univariate time course (I) in which each outcome/condition is analyzed separately; 2) Using a joint multivariate modeling strategy for time course II and III for a) assessing the relation between some covariate and all temporal outcomes simultaneously; b) studying how the association between the various temporal outcomes evolves over time; c) investigating the associations among the evolutions of all temporal outcomes and correlated phenotypes, (e.g., periodical or cycling expression data, time course IV).

Moreover, they are also related to the way the association between and across outcomes is modeled (i.e., with or without latent variables); or how the effects of the variables are treated (random, fixed). So the related approaches can be categorized into classical frequentist inferential approaches (fixed effects), Bayesian models (random effects), or mixed of the classical inferential techniques and Bayesian model, which lead to mixed models [44, 45] (see Fig. 3).

#### Frequentist approaches

Conventional time series techniques such as autoregressive or moving averaging models and Fourier analysis require stationary conditions, linearity for lower order autoregressive models, and uniformly spaced distributed time points, which are not present in short time course omic experiments and therefore are not suitable for unevenly spaced or distributed omic experiment [46, 47]. The repeated analyses of variances (ANOVA), Generalized estimation equation (GEE) or generalized linear mixed models have been applied to time course microarray data. They can model the nonlinear relations between genes, deal with the unevenly time spaced data, and may produce a good fit. But they do not fascinate prediction and may cause over-fitting problems. In addition they do not include the time order information. Functional data analysis methods have been applied to model the temporal data as linear combinations of basis functions (spines) [48, 49].

#### Bayesian methods

The probability and confidence measures play important roles in omic temporal data not only due to the variations, high noise levels and experimental errors resident in the experiments but also the stochastic nature involved in the biological process. The Bayesian paradigm is very well suited for examining these features and other properties in the temporal data, such as highly correlated inputs (genes, time points) and phenotypes, missing data, and small sample size [50–56]. In Bayesian models, the parameters are assumed to be random variables and they are associated with some probability distribution, and the posterior probability of these parameters can be expressed as marginal distribution of those remaining parameters.

Moreover, Bayesian approaches can account for the variability induced by the collection of models and construct credible intervals accounting for model uncertainty through investigating the impact of the choice of priors on model space. Then they can construct new search algorithms that take advantage of parallel processing with Markov Chain Monte Carlo (MCMC) algorithm. Bayesian approaches can be used in the case when there are more covariates than observations. Bayesian method is a hybrid generative-discriminative model that can add prior knowledge (such as distributions of the input) or encode the domain knowledge to improve the learning or training phases. Bayesian approaches can well capture linear, non-linear, combinatorial, and stochastic types of relationships among variables across multiple levels of biological organization and have been extensively applied for the time course gene expression study with various hierarchical settings [41, 42, 47–57].

### Computer sciences approaches

#### Machine learning: unsupervised learning versus supervised classifications

Clustering analyses or unsupervised learning without class labels are the most commonly used methods for time course genomic experiments. These approaches are based on similarity or correlation or distance measures for identification of groups of genes with ‘similar’ temporal patterns of expression, which is a critical step in the analysis of kinetic data given the large number of genes involved [58–66]. Hierarchical clustering with heat map, principal component analysis with scatter plots, or dynamic Bayesian clustering (DBC) approaches are a few popular examples [26–28]. DBC can uncover the underlying temporal structure and enable cluster memberships to change for better understanding the development of complex biological organisms and systems [29].

Supervised clustering or classification approaches incorporate known disease status or the prior known genomic knowledge (e.g., functional annotation tools or publications) as class labels for classifying the genomic temporal patterns and disease/health outcomes [66–70]. Support vector machines (SVM), generalized linear model, discriminant analysis, decision tree, random forest, or neural network are popular examples, which were applied to time course genomic experiments. Semi-supervised learning considers the problem of classification when only a small subset of the observations has corresponding class labels. Vibrational approximations or stochastic variational inference algorithm for semi-supervised learning have also been explored with the omics data and have shown an improved predictive accuracy for the disease/clinical outcomes [71, 72].

#### Discriminative compared with generative approaches

Classification or supervised clustering approaches can be also distinguished as either generative versus discriminative models. Generative approaches learn the joint probability of inputs x (e.g., genes) and output class label y (e.g., normal versus disease status), then make prediction based on the conditional probability obtained through Bayes rules. Naïve Bayesian classifier is a simple example of generative approaches [73] while Bayesian or Gaussian mixture models are more sophisticated [55]; while discriminative approaches directly estimate the conditional probability and learn the direct mapping between the input x to class label y, which is preferred due to many compelling reasons, and a popular example is logistic regression model.

Neural network (feed forward NN, convolutional NN, Bayesian NN) is popular computational approach for prediction problems, which can either apply discriminative or generative strategies [70, 71, 74]. Both unsupervised (i.e., self-organized map) and supervised NN (hierarchical Bayesian NN) have been applied to temporal genomic data for pattern/disease subtype discovery/identifications, or disease classifications/predictions. Neural network with traditional incremental learning and gradient descent algorithms have good classification performance, but such algorithms could be trapped by local minimum solutions when one optimizes a performance/score function, i.e., optimizing the expected reward or minimizing loss functions. In order to find the global optimal solution, recent developed deep learning approach on convolutional NN uses higher order derivative of score functions to obtain the higher order of moments for global optimizations that can handle the convex and local trap issues that may cause misclassifications [75].

### Advanced network and module based approaches

The computational or statistical approaches for network construction include various levels such as transcriptional regulation network, metabolic network, protein-protein, and disease-drug-genes network [76–88]. Networks and module-based approaches reveal hidden patterns in the original unstructured data by transforming raw temporal data into logically structured, clustered, and interconnected graphs [89–95]. These graphs can be visualized with nodes representing genes, proteins and metabolites, and with edges indicating interactions, the potential causal relationships between biological entities (i.e., genes/proteins) or clusters that share similar molecular functions [96–103].

For instance, weighted correlation network analyses identify modules/clusters of highly correlated transcripts, genes, proteins, metabolites [104, 105]. Bayesian network approaches utilize and integrate prior biological domain knowledge (e.g., biochemical pathways, biological processes) with omics data to estimate probabilistic interactions for pathway and biochemical ontology-based integration [106–112]. Friedman and co-workers have used static Bayesian networks, which are graph based models of joint multivariate probability distributions that assess conditional independence between variables. The network obtains simpler sub-models to describe gene interactions from micorarray data [113]. Kimm et al. developed an algorithm to identify interaction network and coupled it with non-parametric regression methods [64].

Dynamic Bayesian networks (DBN) have been popular for learning and inferring the gene regulatory networks, which have been compared with Granger causality and probabilistic Boolean network [43, 106–109, 114–116]. DBN was also combined with other techniques such as Bayesian regularization in order to handle the non-homogeneous, non-stationary and gradually time-varying structure of time course omics data [106, 116].

For examining the potential causal relationships and network structure, autoregressive models for gene regulatory network inference using time course data for sparsity, stability and causality were investigated [117]. Granger causality approach have been developed for genetic network constructions, and applied for measuring the predictive causality of temporal data [57, 114, 118–122]. Furqan and Siyal proposed the LASSO-based Elastic-Net Copula Granger causality for biological network modeling [118]. Their proposed method shows the merits of overcoming high dimensionality issues of ordinary least-squares methods and linear constraints. Marinazzo et al. (2015) propose a kernel Granger causality method for dynamical networks. They address both the nonlinearity (choosing the kernel function) and false causalities issues (selection strategy of the eigenvectors of a reduced Gram matrix). The results showed that the proposed method is a better choice than using L1 minimization methods [57]. However, Granger causality does not account for latent confounding effects and may not be able to capture instantaneous causal relationships [118, 119].

To investigate the dynamic aspects of gene regulatory networks measured through system variables at multiple time points, Acerbi et al. (2014) proposed continuous time Bayesian networks for network reconstruction. They compared two state-of-the-art methods: dynamic Bayesian networks and Granger causality analysis [123]. Results showed that continuous time Bayesian networks were effective on networks of both small and large size, and were particularly feasible when the measurements were not evenly distributed over time. They applied to the reconstruction of the murine Th17 cell differentiation network, and revealed several autocrine loops, suggesting that Th17 cells may be auto regulating their own differentiation process.

### Pathway and function integrative approaches

Two general categories for data integrations are either through meta-analysis (e.g., Venn diagram), which performs analysis for each individual dataset first, then combines the results; or mega-analysis, which combines the data first then conducts the analysis. No matter which strategy, for better interpretations and visualization purposes, pathway and functional analysis need be conducted. The pathway based analysis move to next level of analysis (complementary to the DAVID and KEGG) to define how the selected individually regulated genes, transcripts, or metabolites interact as parts of complex pathways, such as signaling, metabolic pathways based on known knowledge and published literature [124–126].

For instance, using Ingenuity Pathway Analysis software (http://www.ingenuity.com/) that computes a score for each network according to the fit of the network, one can select a cut-off score of 3 for identifying gene networks significantly affected by the specific gene or genotypes. This score indicates that there is a 1/1000 chance that the genes are in a network due to random chance and therefore, scores of 3 or higher have a 99.9% confidence of not being generated by random chance alone. Then one may compare the selected pathways and networks between DEG lists obtained from individual comparisons (allele carrier vs. not) to find the common and unique pathways between each compartment. These comparisons will indicate the difference of specific genes at the pathway level in addition to our biological process and molecular function analyses, pinpointing the relationship among potential candidate driver genes, chromosomal abnormalities, and pathways.

However, biological pathways are inherently complex and dynamic, pathway annotations in different pathway databases vary significantly in pathway models and in a number of other aspects. For instance, specific protein forms, dynamic complex formation, subcellular locations, and pathway cross talks. Interpretation of pathway mapping results from the fact that pathway annotations currently take little consideration of tissue/urine/serum specificities of genes or proteins in the pathway, thus, specific steps of a pathway may not be actually active in tissues/cells from which the data may be generated which is a limitation.

Further function over-representation analysis through the Database for Annotation, Visualization and Integrated Discovery (DAVID; https://david.ncifcrf.gov) identify modules and entities that are enriched and statistically significant over-representation of particular functional categories and major gene/metabolites groups/families [83, 84, 127–129]. Combining with other enrichment and function analysis can facilitate biological interpretation to interrogate complex biological systems for more accurate P4 outcomes [85, 130].

## Applications, software, resources

DREAM (The Dialogue for Reverse Engineering Assessment and Methods project (http://www.the-dream-project.org/) provided excellent examples for temporal omics data sets that involve various most updated biomedical challenge questions (e.g. regulatory network inference, causal inferences, dynamic trajectory predictions) through multiple team competitions [16, 17, 86, 118, 119, 131, 132]. For instance, in DREAM 8 (breast cancer network inference challenge), four breast cancer cell lines were stimulated (under inhibitor perturbations) with eight ligands, which comprised of protein abundance time-courses (from 0 min, to, 5, 15, 30, 60, 120, 240 min) for inferring causal signaling networks and predicting trajectory of protein phosphorylation dynamics in cancer [131]. Inferring a causal network is extremely challenging, which significantly differs from association or correlation network. Constructing the dynamical models that can predict trajectories under specific biological perturbations lead to different signaling responses in different backgrounds is also nontrivial task. Results suggest that learning causal relationships may be feasible in complex settings, such as disease states and incorporating known biology was generally advantageous. For drug prediction challenge, the hybrid, Bayesian multitask approaches, which combines nonlinear regression, multiview learning, multitask learning and Bayesian inference (using prior biological knowledge) has showed best performance for predicting drug response based on a cohort of genomic, epigenomic and proteomic profiling data sets measured in human breast cancer cell lines [132].

Furqan and Siyal (2016) utilized silico temporal gene expression data sets from DREAM4 for inferring network structures and predicting the response of the networks to novel perturbations in an optional “bonus round” [118]. They proposed bi-directional Random Forest Granger causality using the random forest regularization together with the idea of reusing the time series data by reversing the time stamp to extract more causal information. The ensembing approach was applied to HeLa cell dataset to map gene network involved in cancer [119]. From another study, Marinazzo et al. applied Kernel Granger causality using the same data set with 94 genes and 48 time points. Results showed evidence of 19 causal relationships, all involving genes related to tumor development [57].

Eren et al. (2015) developed an advanced automated and human-guided characterization and visualization platform for microbial genomes in metagenomic assemblies. The platform has interactive interfaces that can link omics data from multiple sources into a single, intuitive display [87]. The software includes multi-levels from data preprocessing (i.e., merging, profiling), to unsupervised and supervised learning, hidden Markov model for metagenomics shot read RNA-seq data. They analyzed time course infant gut metagenomes data set (at days 15–19 and 22–24 after birth), and explored temporal genomic changes within naturally occurring microbial populations through de novo characterization of single nucleotide variations. They also linked those with cultivar and single-cell genomes with metagenomic and metatranscriptomic data. They identified systematic emergence of nucleotide variation in an abundant draft genome bin in an infant’s gut. Other applications to different common disease and health conditions by integrations of temporal omics data ranged from single cell analysis to multiple tissues/organs and have been extended by leveraging to social environmental interactions [88, 133–147].

The most popular software packages for conducting computations are omics data are the Bioconductor from R, toolboxes from Matlab, Genomics from SAS/JMP. In addition, C++, Visual Basic, Python, Java, and JavaScript, WinBugs are often used programming languages for developing various types of analytic, visualization tools, pipelines [148–155]. For instance, Bioconductor and R include more than 1290 packages extending the basic functionality of R or connect R to other software, which conduct various types of omics data analysis discussed in section II. More importantly, those packages can incorporate the correlation analysis with other types of relationships such as biochemical reactions and molecular structural and mass spectral similarity (MetaMapR).

In addition, they provide a dynamic interface (Grinn) to integrate gene, protein, and metabolite data using more advanced biological-network-based approaches such as Gaussian graphical models, partial correlation and Bayesian networks for omics data integration (glasso, qpgraph). For instance, time-vaRying enriCHment integrOmics Subpathway aNalysis tOol (CHRONOS) is an R package built to extract regulatory sub-pathways along with their miRNA regulators at each time point based on KEGG pathway maps and user-defined time series mRNA and microRNA (if available) expression profiles for microarray experiments [156, 157]. It can assist significantly in complex disease analysis by enabling the experimentalists to shift from the dynamic to the more realistic time-varying view of the involved perturbed mechanisms. NSPEcT is based on differential equation that describes the process of synthesis and processing of pre-mRNA and the degradation of mature mRNA. It’s a package used for estimation of total mRNA levels, pre-mRNA levels, and degradation rates over time for each gene (from time course RNA-seq) [158]. Furthermore, NSPEcT can test different models of transcriptional regulation to identify the most likely combination of rates explaining the observed changes in gene expression.

Some popular interaction and network analysis resources and databases for biological systems resulted from literatures including IntAct, BioGRID, and MINT. Other network construction software could be useful such as Genetic Network Analyzer (GNA), which is a computer tool for modeling and simulation of gene regulatory networks. GNA allows the dynamics of a gene regulatory network to be analyzed without quantitative information on parameter values, analyzing its dynamical behavior in a qualitative way [159]. For efficient and fast learning the network, Dojer et al. and Wilczyński designed faster Bayesian network learning algorithms and software [160, 161]. Ingenuity Pathway Analysis demonstrates that a module and network based analysis leads to more significant functional enrichment results than a standard analysis based on differential analysis. Table 2 provides some popular platform, software and database links for various types of temporal omics data ranged from fundamental data preprocessing, to immediate analysis to advanced network and pathway and integration analysis.

**Table 2:** Temporal omic data software, libraries and packages, tools and web resources ranged from fundamental data preprocessing, immediate analysis to advanced network and pathway and integration analysis

Software	Omics variety data, formats	Features/Functions/packages	Web links
SAS/JMP Genomics	Various types of genomic data from case-control, SNPs, RNA seq…	Quality-control tools including batch effect removal, PCA, ANOVA, differential analysis, cluster, and prediction e.g., Grinn, MetaMapR, glasso, qpgraph	https://www.jmp.com/en_us/software/data-analysis-software.html
Matlab	Gene-expression, exon-expression, proteins	Neural network, math optimization modeling, nonlinear dynamic systems; prediction,Multidimensional data visualization, Statistical/machine	http://www.mathworks.com/
Bioconductors R	All types of omics data, More than 1200 packages, annotation, experiments, explore, analyze, visualize,	Quality assurance analysis, normalizationVarious statistical (including Bayesian modeling) and algorithm based tools, Cloud-enabled	http://bioconductor.org/
Qlucore Omics Explorer Genedata Expressionist®	RNA seq, microarray, miRNA, Methylation, MS for proteins and metabolites, and Flow cytometry data	Visualization, and biological interpretation; view on the chromatograms; Integration with proteomic and metabolomic data, Automated quality and pre-processing, Standardized workflows	www.qlucore.com https://www.genedata.com/products/expressionist/proteomics/
DNASTAR GENOSTAR	Exon gene level Microarray, NGS, Protein, RNA-Seq, SNP Metagnomics, chip to chip	Visualizing and Comparing, Multiple Genome-Scale Assemblies modelling and simulation of regulatory networks Automated annotation.	http://www.dnastar.com/t-dnastar-lasergene.aspx http://www.genostar.com/category/products/gna/
iPathwayGuide	miRNA Activity, Molecule interactions DNA proteins interactions	Topology-based Analysis Advanced Correction Factors Prediction, Downstream Impact Analysis Meta Analysis	Advaita Bioinformatics: www.advaitabio.com
iBioguide	Genes, microRNAs, pathways, biological processes, molecular functions, cellular components, drugs, diseases,	find related genes, pathways, biological processes, molecular functions, cellular components, drugs, diseases,	https://ibioguide.advaitabio.com/
iVariantGuide Genotyping™ Console Software Clinical Genome nClinGen/ClinVar) Rosetta	SNP, copy number variation, SNP genotyping, indel detection	Analyze rare and common variants Genotyping calls, loss of heterozygosity; Dynamic Graphic Filters Pathway Analysis GO Terms Analysis Cloud-based Sharing, Data management and a data repository	www.advaitabio.com https://genegrid.genomatix.com/grid/home. https://www.clinicalgenome.org/tools/webresources/clinician cross-technology/platform analyses
The Rat Genome Database pathway diagrams	Molecular and physiological pathway; e.g., identifying up or down regulated genes in pathways, see how pathways relate to each other	Pathway acquisition and visualization, multi-layered approach, dynamic and integrated manner, interactive diagram	http://rgd.mcw.edu
Biotique	Next Generation Sequencing Data, XRAY or other expression, FASTA, FASTQ	Excel plug-in interfaces, Integrated annotations, Illumina Genome Analyzer Pipeline	http://www.biotiquesystems.com/Products-Solutions/GenePress- Solutions/GenePress

## Discussion

Learning and integrating dynamic omics temporal data and gene-protein-disease-drug/treatment correlation, interdependence and causal networks between hybrid systems may improve our understanding of system-wide dynamics and errors of pharmacological and biomedical agents and their genetic and environmental modifiers. Most available dynamic approaches and existing applications focus on the genomic time course data, but the same techniques or methodologies can be extended and employed to various types of omics data (such as metagenomics) with the applications to other biological networks and pathways. For instance, RNA-Seq data has revealed far more about the transcriptome than microarrays, primarily because analysis is not limited to known genes. This opens possibly for splicing analysis, analyzing differential allele expression, variant detection, alternative start/stop, gene fusion detection, RNA editing and eQTL mapping.

Either from computational complexity or clinical reproducibility point of view, one cost effective resolutions and future directions would be develop more intelligent AI based data integrations, learning and automations with hierarchical ensemble approaches, not just connectivity. With efficient multi-task learning algorithms (with automatic reasoning and consensus predictions with boosts and bagging) embedded into multilayer computational automated ensemble model systems with pipelines, the latent component of correlated biological entities can be divided and the key components/pathway or elements can be captured through utilizing continuously arriving, evolving, temporal omics data. Investigating the causality rather than the association among various biological entities ranging from RNA, microRNA, DNA, gene, protein, disease, and drug in an integrative perspective would be important, to which relative a few integrative efforts have been dedicated so far.

To overcome other bottleneck issues for omics data that may partially arisen from the biomedical systems’ complexity, that encompasses biological/genetic, behavioral, psychosocial, societal, environmental, systems-related, ethical and other intertwined factors. Further incorporations of electronic health records linked to behavioral, psychosocial, societal, environmental, and clinical lab measures with temporal omics data in hierarchical ensemble automated system will provide us more interpretable and reproducible scientific results and practical clinical decision making for P4 patient outcomes.

## References

[1] McElheny, V. K. (2010). Drawing the Map of Life: Inside the Human Genome Project. Basic Books. ISBN 978-0-465-03260-0.

[2] Tieri P, de la Fuente A, Termanini A (2011). Integrating omics data for signaling pathways, interactome reconstruction, and functional analysis. (2011). Methods Mol Biol.

[3] Carrell DT, Aston KI, Oliva R (2016). The “omics” of human male infertility: integrating big data in a systems biology approach. Cell Tissue Res.

[4] BIG Data Center Members (2017). The BIG Data Center: from deposition to integration to translation. Nucleic Acids Res.

[5] Kim D, Joung JG, Sohn KA (2015). Knowledge boosting: a graph-based integration approach with multi-omics data and genomic knowledge for cancer clinical outcome prediction. J Am Med Inform Assoc.

[6] Barabasi AL, Gulbahce N, Loscalzo J (2011). Network medicine: a network-based approach to human disease. Nat Rev Genet.

[7] Winterbach W, Mieghem P, Reinders M (2013). Topology of molecular interaction networks. BMC Syst Biol.

[8] Vogt H, Hofmann B, Getz L (2016). The new holism: P4 systems medicine and the medicalization of health and life itself. Med Health Care Philos.

[9] Guo NL (2011). Network medicine: New paradigm in the omics Era. Anat Physiol.

[10] Lecca P, Nguyen TP, Priami C (2011). Network inference from time-dependent omics data. Methods Mol Biol.

[11] Machado D, Costa RS, Rocha M (2011). Modeling formalisms in systems biology. AMB Express.

[12] Liang Y, Kelemen A (2006). Associating phenotypes with molecular events: recent statistical advances and challenges underpinning microarray experiments. J Funct Integr Genomics.

[13] Liang, Y., Kelemen, A. (2016). Big Data Science and its Applications in Health and Medical Research: Challenges and Opportunities, Austin Journal of Biometrics & Biostatistics, 7(3). doi: 10.4172/2155-6180.1000307

[14] Kelemen, A., Liang, Y., Vasilakos, A. (2008). Review of Computational Intelligence for Gene-Gene Interactions in Disease Mapping, in “Computational Intelligence in Medical Informatics” (A. Kelemen, A. Abraham, Y. Chen, Eds.) in the Series in Studies in Computational Intelligence, 1-16

[15] Liu YY, Slotine JJ, Barabasi AL (2011). Controllability of complex networks. Nature.

[16] Prill RJ, Marbach D, Saez-Rodriguez J, Sorger PK, Alexopoulos LG, Xue X, Clarke ND, Altan-Bonnet G, Stolovitzky G (2010). Towards a rigorous assessment of systems biology models: the DREAM3 challenges. PLoS One.

[17] Daniel M, Costello JC, Robert K, Nicole V, Prill RJ, Camacho DM, Allison KR (2012). The DREAM5 Consortium, Manolis Kellis, James J Collins, & Gustavo Stolovitzky. Nature Methods.

[18] Grigorov MG (2011). Analysis of time course omics datasets. Methods Mol Biol.

[19] Holter NS, Maritan A, Cieplak M (2001). Dynamic modeling of gene expression data. Proc Natl Acad Sci.

[20] Bar-Joseph Z, Gitter A, Simon I (2012). Studying and modelling dynamic biological processes using time-series gene expression data. Nat Rev Genet.

[21] Bar-Joseph Z, Gerber GK, Gifford DK (2004). Continuous representations of time-series gene expression data. J Comput Biol.

[22] Ramoni MF, Sebastiani P, Kohane IS (2002). Cluster analysis of gene expression dynamics. Proc Natl Acad Sci.

[23] de Jong H, Page M (2008). Search for steady states of piecewise-linear differential equation models of genetic regulatory networks. IEEE/ACM Trans Comput Biol Bioinform.

[24] Davidich M, Bornholdt S (2008). The transition from differential equations to Boolean networks: a case study in simplifying a regulatory network model. J Theor Biol.

[25] Le Novere N (2015). Quantitative and logic modelling of molecular and gene networks. Nat Rev Genet.

[26] Shmulevich, I., Dougherty, E. R. (2010). Probabilistic Boolean networks: The modeling and control of gene regulatory networks, SIAM Press.

[27] Mussel C, Hopfensitz M, Kestler HA (2010). BoolNet-an R package for generation, reconstruction and analysis of Boolean networks. Bioinformatics.

[28] Monteiro PT, Ropers D, Mateescu R (2008). Temporal logic patterns for querying dynamic models of cellular interaction networks. Bioinformatics.

[29] Leek J, (2011) Asymptotic Conditional Singular Value Decomposition for High-Dimensional Genomic Data Biometrics. 67 (2), pp. 344–52. 10.1111/j.1541-0420.2010.01455.x PMC3165001 20560929

[30] Carvalho CM, Chang J, Lucas JE (2008). High-dimensional sparse factor modelling: applications in gene expression genomics. J Am Stat Assoc.

[31] Carvalho CM, West M (2007). Dynamic matrix-variate graphical models. Bayesian Anal.

[32] Carvalho CM, West M, Bernardo JM (2007). Dynamic matrix-variate graphical models—a synopsis. Bayesian statistics.

[33] Peterson C, Stingo F, Vannucci M (2014). Bayesian inference of multiple Gaussian graphical models. J Am Stat Assoc.

[34] Liang F, Song Q, Qiu P (2015). An equivalent measure of partial correlation coefficients for high dimensional Gaussian graphical models. J Am Stat Assoc.

[35] Kossenkov AV, Ochs MF (2009). Matrix factorization for recovery of biological processes from microarray data. Methods Enzymol.

[36] Ramsey S, Orrell D, Bolouri H (2005). Dizzy: stochastic simulation of large-scale genetic regulatory networks. J Bioinform Comput Biol.

[37] Chowdhury AR, Chetty M, Evans R (2015). Stochastic S-system modeling of gene regulatory network. Cogn Neurodyn.

[38] Tanevski J, Todorovski L, Dzeroski S (2016). Learning stochastic process-based models of dynamical systems from knowledge and data. BMC Syst Biol.

[39] Chen KC, Wang TY, Tseng HH (2005). A stochastic differential equation model for quantifying transcriptional regulatory network in Saccharomyces cerevisiae. Bioinformatics.

[40] Swain MT, Mandel JJ, Dubitzky W (2010). Comparative study of three commonly used continuous deterministic methods for modeling gene regulation networks. BMC Bioinform.

[41] Rangel C, Angus J, Ghahramani Z (2004). Modeling T-cell activation using gene expression profiling and state-space models. Bioinformatics.

[42] Yuan M, Kendziorski C (2006). Hidden Markov models for microarray time course data in multiple biological conditions. J Am Stat Assoc.

[43] Perrin BE, Ralaivola L, Mazurie A (2003). Gene networks inference using dynamic Bayesian networks. Bioinformatics.

[44] Durbin J, Koopman SJ (2000). Time series analysis for non-Gaussian observations based on state space models from both classical and Bayesian perspectives (with discussion), J. R Stat Soc, Series B.

[45] Wolfinger RD, Gibson G, Wolfinger ED (2001). Assessing gene significance from cDNA microarray expression data via mixed models. J Comp Biol.

[46] Fujita A, Sato JR, Garay-Malpartida HM, Yamaguchi R, Miyano S, Sogayar MC, Ferreira CE (2007). Modeling gene expression regulatory networks with the sparse vector autoregressive model. BMC Syst Biol.

[47] Ernst J, Nau GJ, Bar-Joseph Z (2005). Clustering short time series gene expression data. Bioinformatics.

[48] de Hoon MJL, Imoto S, Miyano S (2002). Statistical analysis of a small set of time-ordered gene expression data using linear splines. Bioinformatics.

[49] Coffey N, Hinde J (2011). Analyzing time-course microarray data using functional data analysis - a review. Stat Appl Genet Mol Biol.

[50] Mitra R, Müller P, Liang S (2013). A Bayesian graphical model for chip-seq data on histone modifications. J Am Stat Assoc.

[51] Ferrazzi F, Sebastiani P, Ramoni MF (2007). Bayesian approaches to reverse engineer cellular systems: a simulation study on nonlinear Gaussian networks. BMC Bioinform.

[52] Troyanskaya OG, Dolinski K, Owen AB (2003). A Bayesian framework for combining heterogeneous data sources for gene function prediction (in Saccharomyces cerevisiae). Proc Natl Acad Sci U S A.

[53] Liang Y, Kelemen A (2009). Bayesian finite Markov mixture model for temporal multi-tissue polygenic patterns. Biom J.

[54] Liang Y, Kelemen A (2008). Bayesian models and meta analysis for multiple tissue gene expression data following corticosteriod administration. BMC Bioinform.

[55] Liang Y, Kelemen A (2007). Bayesian state space models for inferring and predicting temporal gene expression profiles. Biom J.

[56] Liang Y, Kelemen A (2016). Bayesian state space models for dynamic genetic network construction across multiple tissues. J Stat Appl Genet Mol Biol.

[57] Marinazzo D, Pellicoro M, Stramaglia S (2008). Kernel-Granger causality and the analysis of dynamical networks. Phys Rev E Stat Nonlin Soft Matter Phys.

[58] Gasch, A. P., Eisen, M. B. (2002). Exploring the conditional coregulation of yeast gene expression through fuzzy k-means clustering. Genome Biology 3(11). 10.1186/gb-2002-3-11-research0059 PMC133443 12429058

[59] Huang, H., Cai, L., Wong, W. H. (2008). Clustering analysis of SAGE transcription profiles using a Poisson approach. in SAGE: Methods and Protocols, ed. K. L. Nielsen, Humana Press Inc. 10.1007/978-1-59745-454-4_14 18287632

[60] Eisen MB, Spellman PT, Brown PO, Botstein D (1998). Cluster analysis and display of genome-wide expression patterns. Proc Natl Acad Sci.

[61] D’haeseleer P (2005). How does gene expression clustering work?. Nat Biotechnol.

[62] Yeung KY, Ruzzo WL (2001). Principal component analysis for clustering gene expression data. Bioinformatics.

[63] Tamayo P, Slonim D, Mesirov J (1999). Interpreting patterns of gene expression with self-organizing maps: methods and application to hematopoietic differentiation. Proc Natl Acad Sci U S A.

[64] Fowler A, Menon V, Heard NA (2013). Dynamic Bayesian clustering. J Bioinform Comput Biol.

[65] D’haeseleer P, Liang S, Somogyi R (2000). Genetic network inference: from co expression clustering to reverse engineering. Bioinformatics.

[66] Dettleing, M. and Bühlmann, P. (2002). Supervised clustering of genes. Genome Biology. 3:research0069.1-0069.15. 10.1186/gb-2002-3-12-research0069 PMC151171 12537558

[67] Zhang Y, Tibshirani R, Davis R (2013). Classification of patients from time-course gene expression. Biostatistics.

[68] Komura D, Nakamura H, Tsutsumi S (2005). Multidimensional support vector machines for visualization of gene expression data. Bioinformatics.

[69] Liang Y, Kelemen A (2009). Time lagged recurrent neural network for temporal gene expression classification. Int J Comput Intell Bioinform Syst Biol.

[70] Liang Y, Kelemen A (2005). Temporal gene expression classification with regularised neural network. Int J Bioinform Res Appl.

[71] Xu R, Wang Q (2013). A semi-supervised pattern-learning approach to extract Pharmacogenomics-specific drug-gene pairs from biomedical literature. J Pharmacogenom Pharmacoproteomics.

[72] Shi M, Zhang B (2011). Semi-supervised learning improves gene expression-based prediction of cancer recurrence. Bioinformatics.

[73] Kelemen A, Zhou H, Lawhead P (2003). Naive Bayesian classifier for microarray data. IEEE Proc Int Jt Conf Neural Netw.

[74] Liang Y, Kelemen A (2004). Hierarchical Bayesian neural network for gene expression temporal patterns. J Stat Appl Genet Mol Biol.

[75] Peng H-K, Marculescu R (2015). Multi-scale compositionality: identifying the compositional structures of social dynamics using deep learning. PLoS One.

[76] Yu J, Smith VA, Wang PP (2004). Advances to Bayesian network inference for generating causal networks from observational biological data. Bioinformatics.

[77] Karlebach G, Shamir R (2008). Modelling and analysis of gene regulatory networks. Nat Rev Mol Cell Biol.

[78] Marbach D, Costello JC, Küffner R (2012). Wisdom of crowds for robust gene network inference. Nat Methods.

[79] Saris CGJ, Horvath S, van Vught PWJ (2009). Weighted gene co-expression network analysis of the peripheral blood from amyotrophic lateral sclerosis patients. BMC Genomics.

[80] Ghasemi O, Lindsey ML, Yang T (2011). Bayesian parameter estimation for nonlinear modeling of biological pathways. BMC Syst Biol.

[81] Boué, S., Talikka, M., Westra, J. W., et al. (2015). Causal biological network database: a comprehensive platform of causal biological network models focused on the pulmonary and vascular systems. Database. Article ID bav030. 10.1093/database/bav030 PMC4401337 25887162

[82] Cerami E, Demir E, Schultz N (2010). Automated network analysis identifies core pathways in glioblastoma. PLoS One.

[83] Jang Y, Yu N, Seo J (2016). MONGKIE: an integrated tool for network analysis and visualization for multi-omics data. Biol Direct.

[84] Hecker M, Lambeck S, Toepfer S (2009). Gene regulatory network inference: data integration in dynamic models-a review. Biosystems.

[85] Junker BH, Klukas C, Schreiber F (2006). VANTED: a system for advanced data analysis and visualization in the context of biological networks. BMC Bioinforma.

[86] Noren DP, Long BL, Norel R, Rrhissorrakrai K, Hess K (2016). A Crowdsourcing approach to developing and assessing prediction algorithms for AML prognosis. PLoS Comput Biol.

[87] Eren AM, Esen ÖC, Quince C, Vineis JH, Morrison HG, Sogin ML, Delmont TO (2015). Anvi’o: an advanced analysis and visualization platform for ‘omics data. PeerJ.

[88] Setty M, Tadmor MD, Reich-Zeliger S, Angel O, Salame TM, Kathail P, Choi K, Bendall S, Friedman N, Pe’er D (2016). Wishbone identifies bifurcating developmental trajectories from single-cell data Nat. Biotech.

[89] Litvin O, Causton H, Chen BJ, Pe’er D (2009). Modularity and interactions in the genetics of gene expression. Proc Natl Acad Sci.

[90] Marbach D, Schaffter T, Mattiussi C (2009). Generating realistic in silico gene networks for performance assessment of reverse engineering methods. J Comput Biol.

[91] Marbach D, Prill RJ, Schaffter T, Mattiussi C, Floreano D, Stolovitzky G (2010). Revealing strengths and weaknesses of methods for gene network inference. Proc Natl Acad Sci U S A.

[92] Marbach D, Schaffter T, Mattiussi C, Floreano D (2009). Generating realistic “in silico” gene networks for performance assessment of reverse engineering methods. J Comput Biol.

[93] Segal E, Shapira M, Regev A (2003). Module networks: identifying regulatory modules and their condition-specific regulators from gene expression data. Nat Genet.

[94] Pal R, Bhattacharya S (2012). Transient dynamics of reduced-order models of genetic regulatory networks. IEEE/ACM Trans Comput Biol Bioinform.

[95] Wang YK, Hurley DG, Schnell S (2013). Integration of steady-state and temporal gene expression data for the inference of gene regulatory networks. PLoS One.

[96] Wang J, Chen G, Li M (2011). Integration of breast cancer gene signature based on graph centrality. BMC Syst Biol.

[97] Foster DV, Kauffman SA, Socolar JES (2006). Network growth models and genetic regulatory networks. Phys Rev E.

[98] Yu H, Kim PM, Sprecher E (2007). The importance of bottlenecks in protein networks: correlation with gene essentiality and expression dynamics. PLoS Comput Biol.

[99] Ideker, T., Krogan, N. J. (2012). Differential network biology. Mol Syst Biol. 8(565). doi: 10.1038/msb.2011.99 10.1038/msb.2011.99 PMC3296360 22252388

[100] Bhardwaj N, Kim PM, Gerstein MB (2010). Rewiring of transcriptional regulatory networks: Hierarchy, rather than connectivity, better reflects the importance of regulators. Sci Signal.

[101] Kourmpetis YAI, van Dijk ADJ, Bink MCAM (2010). Bayesian Markov random field analysis for protein function prediction based on network data. PLoS One.

[102] Yao C, Li H, Zhou C (2010). Multi-level reproducibility of signature hubs in human interactome for breast cancer metastasis. BMC Syst Biol.

[103] Sophie Lèbre, Jennifer Becq, Frédéric Devaux, Michael PH Stumpf, Gaëlle Lelandais (2010) Statistical inference of the time-varying structure of gene-regulation networks BMC Systems Biology,,4 (1) 10.1186/1752-0509-4-130 PMC2955603 20860793

[104] Carter SL, Brechbühler CM, Griffin M (2004). Gene co-expression network topology provides a framework for molecular characterization of cellular state. Bioinformatics.

[105] Langfelder P, Horvath S (2008). WGCNA: an R package for weighted correlation network analysis. BMC Bioinform.

[106] Dondelinger F, Lèbre S, Husmeier D (2013). Non-homogeneous dynamic Bayesian networks with Bayesian regularization for inferring gene regulatory networks with gradually time-varying structure. Mach Learn.

[107] Dojer N, Gambin A, Mizera A (2006). Applying dynamic Bayesian networks to perturbed gene expression data. BMC Bioinform.

[108] Zou M, Conzen SD (2005). A new dynamic Bayesian network (DBN) approach for identifying gene regulatory networks from time course microarray data. Bioinformatics.

[109] Li P, Zhang CY, Perkins EJ (2007). Comparison of probabilistic Boolean network and dynamic Bayesian network approaches for inferring gene regulatory networks. BMC Bioinform.

[110] Grzegorczyk M, Husmeier D (2011). Non-homogeneous dynamic Bayesian networks for continuous data. Mach Learn.

[111] Wilkinson DJ (2011). Stochastic modelling for systems biology.

[112] Whiteley N, Andrieu C, Doucet A (2010). Efficient Bayesian inference for switching state-space models using discrete particle Markov chain Monte Carlo methods. ArXiv e-prints.

[113] Friedman N, Inferring cellular networks using probabilistic graphical models Carvalho, C. M., West, M (2007). Dynamic matrix-variate graphical models. Bayesian Anal.

[114] Zou C, Feng H (2009). Granger causality vs. Dynamic Bayesian network inference: a comparative study. BMC Bioinform.

[115] Kimm SY, Imoto S, Miyano S (2002). Dynamic Bayesian network and nonparametric regression model for inferring gene networks. Genome Inform.

[116] Robinson J, Hartemink A (2010). Learning Non-stationary dynamic Bayesian networks. J Mach Learn Res.

[117] Michailidis G, d‘Alché-Buc F (2013). Autoregressive models for gene regulatory network inference: sparsity, stability and causality. Math Biosci.

[118] Furqan MS, Siyal MY (2016). Elastic-Net copula granger causality for inference of biological networks. PLoS One.

[119] Furqan MS, Siyal MY (2016). Inference of biological networks using Bi-directional random forest granger causality. Springerplus.

[120] Tam GH, Chang C, Hung YS (2013). Gene regulatory network discovery using pairwise granger causality. ET Syst Biol.

[121] Yao S, Yoo S, Yu D (2015). Prior knowledge driven granger causality analysis on gene regulatory network discovery. BMC Bioinform.

[122] Lozano AC, Abe N, Liu Y, Rosset S (2009). Grouped graphical granger modeling for gene expression regulatory networks discovery. Bioinformatics.

[123] Acerbi E, Zelante T, Narang V, Stella F (2014). Gene network inference using continuous time Bayesian networks: a comparative study and application to Th17 cell differentiation. BMC Bioinform.

[124] Kandasamy K, Mohan SS, Raju R (2010). NetPath: a public resource of curated signal transduction pathways. Genome Biol.

[125] Yu N, Seo J, Rho K (2012). hiPathDB: a human-integrated pathway database with facile visualization. Nucleic Acids Res.

[126] Bader GD, Cary MP, Sander C (2006). Pathguide: a pathway resource list. Nucleic Acids Res.

[127] Huang DW, Sherman BT, Lempicki RA (2009). Systematic and integrative analysis of large gene lists using DAVID Bioinformatics Resources. Nature Protoc.

[128] Huang DW, Sherman BT, Lempicki RA (2009). Bioinformatics enrichment tools: paths toward the comprehensive functional analysis of large gene lists. Nucleic Acids Res.

[129] Kilicoglu H, Shin D, Fiszman M (2012). SemMedDB: a PubMed-scale repository of biomedical semantic predications. Bioinformatics.

[130] Hu Z, Hung JH, Wang Y, Chang YC, Huang CL, Huyck M, DeLisi C (2009). VisANT 3.5: multi-scale network visualization, analysis and inference based on the gene ontology. Nucleic Acids Res.

[131] Hill SM, Heiser LM, Cokelaer T, Unger M, Nesser NK, Carlin DE, Zhang Y, Sokolov A, Paull EO, Wong CK, Graim K, Bivol A, Wang H, Zhu F, Afsari B, Danilova LV, Favorov AV, Lee WS, Taylor D, Hu CW, Long BL, Noren DP, Bisberg AJ, The HPN-DREAM Consortium, Mills GB, Gray JW, Kellen M, Norman T, Friend S, Qutub AA, Fertig EJ, Guan Y, Song M, Stuart JM, Spellman PT, Koeppl H, Stolovitzky G+, Saez-Rodriguez J+ & Mukherjee S+ (2016). Inferring causal molecular networks: empirical assessment through a community-based effort. Nat Methods.

[132] Costello J, Heiser L (2014). A community effort to assess and improve drug sensitivity prediction algorithms. Nat Biotechnol.

[133] Cambiaghi, A., Ferrario, M., Masseroli, M. (2016). Analysis of metabolomic data: tools, current strategies and future challenges for omics data integration. Briefings in Bioinformatics pii: bbw031. 10.1093/bib/bbw031 27075479

[134] Lei L, Tibiche C, Fu C (2012). The human phosphotyrosine signaling network: evolution and hotspots of hijacking in cancer. Genome Res.

[135] Gut G, Tadmor MD, Pe’er D, Pelkmans L, Liberali P (2015). Trajectories of cell-cycle progression from fixed cell populations. Nat Methods.

[136] Gagneur J, Stegle O, Zhu C, Jakob P, Tekkedil MM, Aiyar RS, Schuon AK, Pe’er D, Steinmetz LM (2013). Genotype-environment interactions reveal causal pathways that mediate genetic effects on phenotype. PLoS Genet.

[137] Wang J, Qiu X, Deng Y (2011). A transcriptional dynamic network during Arabidopsis thaliana pollen development. BMC Syst Biol.

[138] Jia P, Kao CF, Kuo PH (2011). A comprehensive network and pathway analysis of candidate genes in major depressive disorder. BMC Syst Biol.

[139] Xie L, Weichel B, Ohm JE (2011). An integrative analysis of DNA methylation and RNA-Seq data for human heart, kidney and liver. BMC Syst Biol.

[140] Kim W, Li M, Wang J (2011). Biological network motif detection and evaluation. BMC Syst Biol.

[141] Martin G, Marinescu MC, Singh DE (2011). Leveraging social networks for understanding the evolution of epidemics. BMC Syst Biol.

[142] Garcia-Alcalde F, Garcia-Lopez F, Dopazo J, Conesa A (2011). Paintomics a web based tool for the joint visualization of transcriptomics and metabolomics data. Bioinformatics.

[143] Durruthy, R & Heller, S (2015). Applications for single cell trajectory analysis in inner ear development and regeneration. Cell and Tissue Research, 361(1), 49–7. http://doi.org/10.1007/s00441-014-2079-2. 10.1007/s00441-014-2079-2 PMC4480215 25532874

[144] Simidjievski N, Todorovski L, Dzeroski S (2016). Modeling dynamic systems with efficient ensembles of process-based models. PLoS One.

[145] Garcia-Alcalde F, Garcia-Lopez F, Dopazo J, Conesa A (2011). Paintomics: a web based tool for the joint visualization of transcriptomics and metabolomics data. Bioinformatics.

[146] Trauger SA, Kalisak E, Kalisiak J, Morita H, Weinberg MV, Menon AL, Ii Poole FL, Adams MWW, Siuzdak G (2008). Correlating the transcriptome, proteome, and Metabolome in the environmental adaptation of a Hyperthermophile. J Proteome Res.

[147] Sperisen P, Cominetti O, Martin F-PJ (2015). Longitudinal omics modeling and integration in clinical metabonomics research: challenges in childhood metabolic health research. Front Mol Biosci.

[148] Karnovsky A, Weymouth T, Hull T, Tarcea VG, Scardoni G, Laudanna C, Sartor MA, Stringer KA, Jagadish HV, Burant C (2012). Metscape 2 bioinformatics tool for the analysis and visualization of metabolomics and gene expression data. Bioinformatics.

[149] Pavlopoulos G, O’Donoghue S, Satagopam V, Soldatos T, Pafilis E, Schneider R (2008). Arena3D: visualization of biological networks in 3D. BMC Syst Biol.

[150] Shannon P, Markiel A, Ozier O, Baliga NS, Wang JT, Ramage D, Amin N, Schwikowski B, Ideker T (2003). Cytoscape: a software environment for integrated models of biomolecular interaction networks. Genome Res.

[151] McGuffin MJ, Jurisica I (2009). Interaction techniques for selecting and manipulating subgraphs in network visualizations. IEEE Trans Vis Comput Graph.

[152] Barsky A, Munzner T, Gardy J, Kincaid R: C (2008). Visualizing multiple experimental conditions on a graph with biological context. IEEE Trans Vis Comput Graph.

[153] Yordanov, B., Dunn, S. J., Kugler, H., et al. (2016). A method to identify and analyze biological programs through automated reasoning. NP J Systems Biology and Applications. Article number: 16010. 10.1038/npjsba.2016.10 PMC5034891 27668090

[154] Fertig EJ, Stein-O’Brien G, Jaffe A (2014). Pattern identification in time-course gene expression data with the CoGAPS matrix factorization. Methods Mol Biol.

[155] Fertig EJ, Ding J, Favorov AV (2010). CoGAPS: an R/C++ package to identify patterns and biological process activity in transcriptomic data. Bioinformatics.

[156] Vrahatis AG, Dimitrakopoulou K, Balomenos P, Tsakalidis AK, Bezerianos A (2015). CHRONOS: a time-varying method for microRNA-mediated sub-pathway enrichment analysis. Bioinformatics.

[157] Kanehisa M, Goto S, Sato Y (2012). KEGG for integration and interpretation of large-scale molecular datasets. Nucleic Acids Res.

[158] de Pretis S, Kress T, Morelli MJ, Melloni GE, Rival L, Amati B, Pelizzola M (2015). INSPEcT: a computational tool to infer mRNA synthesis, processing and degradation dynamics from RNA- and 4sU-seq time course experiments. Bioinformatics.

[159] Batt G, Besson B, Ciron PE, van Helden J, Toussaint A, Thieffry D (2012). Genetic network analyzer: a tool for the qualitative modeling and simulation of bacterial regulatory networks. Bacterial molecular networks : methods and protocols, methods in molecular biology.

[160] Dojer N, Bednarz P, Podsiadło A (2013). BNFinder2: faster Bayesian network learning and Bayesian classification. Bioinformatics.

[161] Wilczynski B, Dojer N (2009). BNFinder: exact and efficient method for learning Bayesian networks. Bioinformatics.

[162] Villa S, Stella F (2016). Learning continuous time Bayesian networks in Non-stationary domains. J Artif Intel Res.

